# Parasitic causes of meat and organs in cattle at four slaughterhouses in Sistan‐Baluchestan Province, Southeastern Iran between 2008 and 2016

**DOI:** 10.1002/vms3.475

**Published:** 2021-03-15

**Authors:** Javad Khedri, Mohammad Hossein Radfar, Behzad Nikbakht, Rouhollah Zahedi, Mehdi Hosseini, Mohammad Azizzadeh, Hassan Borji

**Affiliations:** ^1^ Department of Pathobiology School of Veterinary Medicine Bahonar University of Kerman Kerman Iran; ^2^ Zahedan Veterinary Office, Sistan and Baluchestan Provincial Veterinary Service Iranian Veterinary Organization Zahedan Iran; ^3^ Department of Clinical Science School of Veterinary Medicine Ferdowsi University of Mashhad Mashhad Iran; ^4^ Department of Pathobiology School of Veterinary Medicine Ferdowsi University of Mashhad Mashhad Iran

**Keywords:** abattoir, cattle, liver, lung, prevalence, Sistan‐Baluchestan Province

## Abstract

This 8‐year (from 2008 to 2016) retrospective study calculated the percentage of carcass and organ (lung and liver) condemnations and estimated the direct financial costs at four slaughterhouses in Sistan‐Baluchestan Province, Southeastern Iran. Each carcass and organ (lung and liver) was thoroughly examined through inspection, palpation and incision following the standard protocol. Identification of the parasites was performed macroscopically. The total direct economic loss due to meat's condemnation was estimated by adding weights of each organ or carcass part and multiplying individual organ totals by their 2016 market unit price. A total of 857,039 cattle were slaughtered during this period, 64,497 livers (7.5%), 31,401 lungs (3.6%) and the carcasses of 1,171 cattle (0.1%) were condemned due to lesions caused by parasites. The main parasitic lesions in the condemned livers were attributed to *Echinococcus granulosus* (4.2%), *Fasciola* spp. (3.1%) and *Dicrocoelium dendriticum* (0.1%). All the condemned lungs were due to *E. granulosus* (3.6%). *Taenia saginata* cysticerci were detected in 0.1% of inspected animals. Liver condemnation due to cystic echinococcosis was the highest in fall (4.7%, *p* < 0.001); while lung condemnation was the highest during spring (3.98%, *p* < 0.001). Liver condemnation due to *Fasciola* spp. was the lowest in winter (2.99%, *p* < 0.001). Carcass condemnation as a result of cysticercosis was the highest in summer (*p* < 0.001). Considering the 2016 market prices, condemnations due to the studied parasites caused direct costs estimated U.S. $ 3,191,879. To the best of our knowledge, this is the first report estimating the monetary losses due to parasitic infections in the slaughterhouses of this province. Due to the high financial impact of the studied parasites, a control programme should be implemented to decrease this impact.

## Graphical abstract

Among the organs examined for the existence of parasitic lesions, 64,497 livers (7.5%), 31,401 lungs (3.6%) and the carcasses of 1,171 cattle (0.13%) were discarded due to parasitic infections. The main parasitic lesions detected in the condemned livers were attributed to *Echinococcus granulosus* (4.2%), *Fasciola hepatica* (3.1%) and *Dicrocoelium dendriticum* (0.1%).

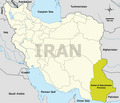

## 1 INTRODUCTION

Meat inspection is one of the most important procedures ensuring the delivery to markets of safe food and enable countries to guarantee the safety and quality of their foods entering international trade (Jaja et al., 2016). During the last two decades, the population of Iran increased from around 66.13 to about 83 million (Iranian Civil Registration Organization, 2019) leading to a significant increase in demand for food specially, animal proteins. Cattle, sheep, goats, camels and their products are the main source of red meat for Iran (Samkange et al., 2019). The slaughterhouse is a relevant occasion for screening infections, mainly those causing zoonotic diseases. Zoonotic infections such as tuberculosis, cysticercosis and cystic echinococcosis, in addition to causing economic losses, are also important in public health.

Iranian Sistani and Brahman cattle breeds are an important and integral part of agricultural production in the Sistan‐Baluchestan Province, Iran. Iranian Sistani and Brahman cattle are an important dual‐purpose breed in eastern Iran (Khedri et al., 2014, 2016). This breed developed distinctive characteristics in response to environmental constraints such as local feed resources, heat and high parasite burdens (Khedri et al., 2014). Scanty information is available on their parasitic infestation in this Province (Khedri et al., 2014, 2015, 2016; Nabavi et al., 2014). Studies on meat condemnation at slaughterhouses have paid less attention to parasites infectionsand have largely and geographically, been restricted to Southwest (Borji, Azizzadeh, & Kamelli, 2012) and north Iran (Borji, Azizzadeh, & Afsai, 2012; Borji & Parandeh, 2010; Jahed Khaniki et al., 2013). Moreover emphasis has been placed on small ruminants (Borji, Azizzadeh, & Afsai, 2012; Jahed Khaniki et al., 2013), small sample sizes of mostly dairy farms or only specific subgroups of animals.

The aim of the current study was to provide baseline data on the common causes of slaughterhouse condemnations of livers, lungs and carcass in a meat inspection survey at four slaughterhouses in the Sistan and Baluchestan Province, Iran. The financial losses due of these condemnations were also estimated for the whole period.

## 2 MATERIALS AND METHODS

**FIGURE 1:**
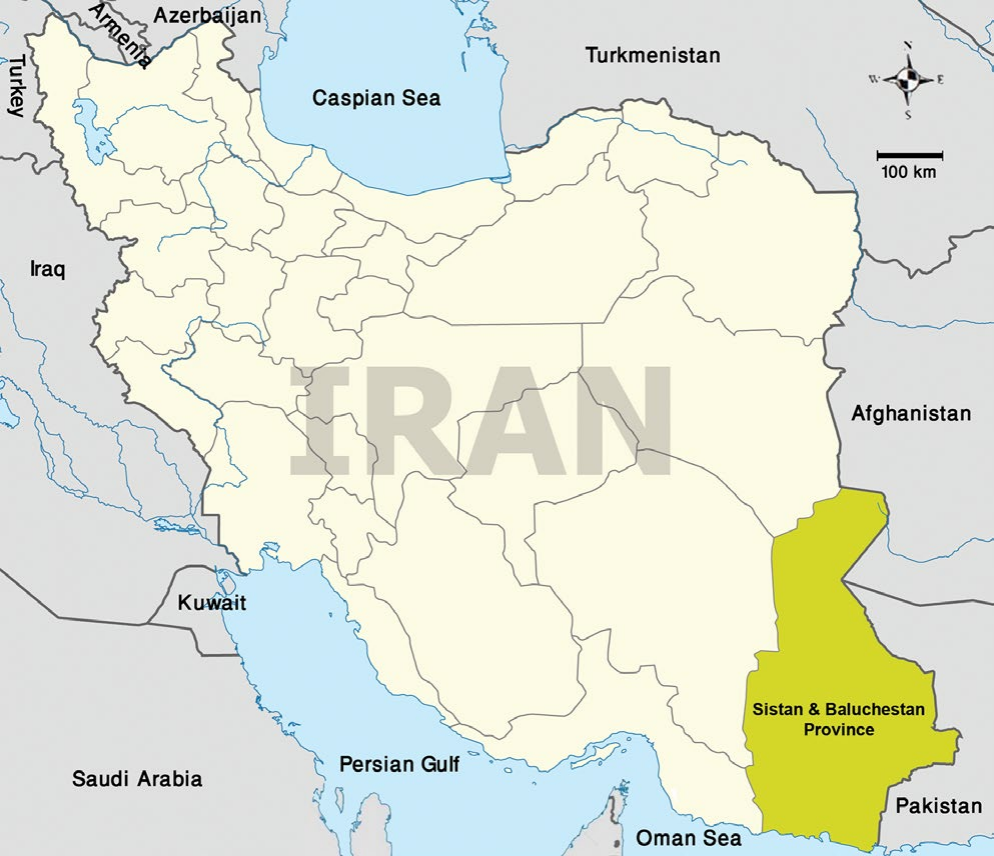
Map of Sistan and Baluchistan Province, Iran, Districts and geographical outlines are depicted

This study was conducted in Sistan and Baluchestan Province, Iran with a geographical area of 180,726 km^2^ and 2.5 million inhabitants. This Province is bordered by Pakistan in the east and Afghanistan in the northeast, by the Southern Khorasan Province in the north; by Kerman province in the west and by the Gulf of Oman in the south. Geographically, the Province consists of two land structures. In the North, Dasht‐ e‐ Sistan formed by Hirmand alluvium holds the largest fresh water lake in the country. The Southern region is mostly mountainous with a variety of climates due to the vicinity of Taftan Volcano and Oman Sea (Figure 1).

A retrospective study was carried out to collect data for 8‐years period (20 April 2008 to 19 March 2016) record in four slaughterhouses in Sistan and Baluchestan Province, South–eastern Iran. Record of meat‐inspection from these slaughterhouses were used to collect information lung, liver and carcass condemnations due to parasitic infections. Each carcass and organ (lung and liver) was thoroughly examined through inspection, palpation and incision following the standard protocol. Costs due to the parasite‐related condemnations over the 8‐year study period were estimated based on2016 market price of carcass, liver and lungs of cattle (Borji, Azizzadeh, & Kamelli, 2012; Borji & Parandeh, 2010).

Statistical analyses were performed using IBM SPSS software package (V.21; IBM Corporation, Armonk, NY, USA). Association of season and year of observation with rate of parasitic infestation were evaluated by chi‐square test. The threshold value of different statistical tests was ≤0.05.

## 3 RESULTS

During the 8‐year period, 857,039 cattle were slaughtered at the slaughterhouses of Sistan‐Baluchestan Province, Southeastern Iran. The livers of 64,497 (7.5%), the lungs of 31,401 (3.6%) and the carcasses of 1,171 (0.1%) cattle were condemned due to parasitic infections.

Hydatid cysts (HC)The condemnation rate of lungs due to HC was 3.6% of them were HC (Table 1). The annual rate of lung condemnations attributable to HC decreased from 6.3% to 4.4% from 2008 to 2016 (*p* < 0.001), respectively. The rate of condemned livers (4.2%) due to HC was significantly higher than the lungs (3.7%) (*p* < 0.001). This rate significantly decreased from 4.5% to 3.3% between 2008 and 2016 (*p* < 0.001), respectively. Liver condemnation rate due to HC was highest in fall (4.7%, *p* < 0.001); while lung condemnation rate was highest in spring (3.98%, *p* < 0.001) (Table 2). The rate of liver and lung condemnation for HC in cattle significantly decreased between 2008 and 2014, while it increased between 2014 and 2015 (*p* < 0.001).

**TABLE 1:** Liver and lung condemned due to *Echinococcus granulosus* infection and liver condemned due to liver fluke in Sistan‐Baluchestan Province, Iran, from 20 April 2008 to 19 March 2016

Year	No. of slaughtered cattle	Number of *Echinococcus granulosus* Infected Livers (%±*SE*)	Number of *Echinococcus granulosus* Infected lungs (%±*SE*)	Number of *Fasciola* spp. infected animals (%±*SE*)	Number of *Dicrocoelium dendriticum* infected animals (%±*SE*)
2008–2009	93,434	4,254 (4.6 ± 0.068)	5,960 (6.4 ± 0.80)	2,471 (2.6 ± 0.052)	45 (0.05 ± 0.007)
2009–2010	124,345	8,637 (6.9 ± 0.072)	4,332 (3.5 ± 0.052)	6,749 (5.4 ± 0.064)	53 (0.04 ± 0.006)
2010–2011	111,754	5,628 (5.0 ± 0.065)	3,458 (3.1 ± 0.052)	4,764 (4.3 ± 0.60)	76 (0.07 ± 0.008)
2011–2012	79,598	2,795 (3.5 ± 0.056)	2,828 (3.6 ± 0.066)	2,211 (2.8 ± 0.058)	129 (0.16 ± 0.014)
2012–2013	115,859	3,110 (2.7 ± 0.047)	4,175 (3.6 ± 0.055)	2,579 (2.2 ± 0.043)	111 (0.10 ± 0.009)
2013–2014	134,612	3,585 (2.7 ± 0.044)	2,519 (1.9 ± 0.037)	2010 (1.5 ± 0.033)	116 (0.09 ± 0.008)
2014–2015	75,410	4,283 (5.7 ± 0.084)	2,659 (3.5 ± 0.067)	3,014 (4.0 ± 0.071)	256 (0.34 ± 0.021)
2015–2016	122,027	4,065 (3.3 ± 0.051)	5,470 (4.5 ± 0.059)	3,455 (2.8 ± 0.047)	101 (0.08 ± 0.008)
Total	857,039	36,357 (4.2 ± 0.022)	31,401 (3.7 ± 0.020)	27,253 (3.2 ± 0.019)	887 (0.1 ± 0.003)

Abbreviation: *SE*, Standard error.

**TABLE 2:** Seasonal variation of Fasciolosis, Dicrocoelium, Liver hydatid cyst and Lung hydatid cyst for the number of liver and lung condemnations in Sistan‐Baluchestan Province, Iran, from 20 April 2008 to 19 March 2016

Seasons	Slaughtered	Liver hydatid cyst			Lung hydatid cyst			*Dicrocoelium dendriticum*			*Fasciola* spp.		
	*N*	*n*	% (*SE*)	*p* value	*n*	%(*SE*)	*p* value	*n*	%(*SE*)	*p* value	*n*	% (*SE* ^a^)	*p* value
Spring	220,423	9,450	4.29^a^ (0.04)	<0.001	8,769	3.98^a^ (0.04)	<0.001	220	0.10^a^ (0.007)	<0.001	7,192	3.26^a^ (0.04)	<0.001
Summer	234,843	9,562	4.07^b^ (0.04)		8,950	3.81^b^ (0.04)		289	0.12^a^ (0.007)		7,682	3.27^a^ (0.04)	
Autumn	183,747	8,641	4.70^c^ (0.05)		6,874	3.74^b^ (0.04)		198	0.11^a^ (0.008)		5,859	3.19^a^ (0.04)	
Winter	218,026	8,704	3.99^b^ (0.04)		6,808	3.12^c^ (0.04)		140	0.06^b^ (0.005)		6,520	2.99^b^ (0.04)	

Abbreviation: *SE*, Standard error.

^abc^ Prevalence (%) of infection within a column with different superscripts are significantly different.

### 3.1 *Fasciola* spp.

The condemnation rate of inspected livers for *Fasciola* spp. was 3.2% (Table 1). Between 2008 and 2016, this rate increased from 2.6% to 2.8%, respectively (*p* < 0.001). Condemnation of livers was significantly lower in winter compared with other seasons (*p* < 0.001), similarly, condemnation due to *Fasciola* spp. was lower in winter (2.99% *p* < 0.001) (Table 2).

### 3.2 Dicrocoelium dendriticum

The condemnation rate due to *D. dendriticum* of livers was 0.1% (Table 1). Between 2008 and 2016, this rate increased from 0.04% to 0.08%, respectively (*p* < 0.001). Moreover condemnation rate due to *Fasciola* spp. and *D. dendriticum* decreased during the study period (*p* < 0.001) (Table 1).

### 3.3 *Taenia saginata* cysticerci

The overall rate of condemned carcasses due to *T. saginata* cysticercus infection was estimated to 0.1% (Table 3). Carcasses condemnation due to *T. saginata* cysticercus infection was statistically highest in summer (*p* < 0.001).

**TABLE 3:** Meat condemned due to cysticercosis in Sistan‐Baluchestan Province, Iran, from 20 April 2008 to 19 March 2016

Year	No. of slaughtered cattle	Number of *Taenia saginata* cysticerci infected animals (%±*SE*)
2008–2009	93,434	116 (0.12 ± 0.011)
2009–2010	124,345	135 (0.11 ± 0.009)
2010–2011	111,754	75 (0.07 ± 0.008)
2011–2012	79,598	61 (0.08 ± 0.010)
2012–2013	115,859	71 (0.06 ± 0.007)
2013–2014	134,612	222 (0.16 ± 0.011)
2014–2015	75,410	288 (0.38 ± 0.022)
2015–2016	122,027	203 (0.17 ± 0.012)
Total	857,039	1,171 (0.014 ± 0.004)

Abbreviation: *SE*, Standard error.

Condemnation carcass of cattle due to *T. saginata* cysticerci rate decreased from 0.12 to 0.06 between 2008 and 2013 and then increased during the rest of study period (*p* < 0.001) (Table 3).

### 3.4 Economic losses

Based on 2016 market prices, the whole cost of the meat and the overall loss as a consequence of parasite‐related condemnation in the study regions over the 8‐year period was estimated at U.S $3,191,879. Of this, U.S. $2,937,727 was related to livers’ condemnation, U.S. $78,502 to lungs’ condemnation and U.S. $175,650 to carcasses’ condemnation (Table 4).

**TABLE 4:** Estimated cost of condemnation (U.S. $) due to helminth infection in slaughtered cattle in the slaughterhouse of Sistan‐Baluchestan Provine, Iran, from 20 April 2008 to 19 March 2016

Year	*Echinococcus granulosus*	*Fasciola* spp.	*Dicrocoelium dendriticum*	*Taenia saginata*	Overall Costs
2008–2009	288,582 (86)	163,111 (35)	31,367 (27)	144,296 (31)	627,353 (65)
2009–2010	319,800 (67)	182,718 (46)	40,555 (24)	170,805 (18)	713,878 (46)
2010–2011	248,126 (45)	110,195 (28)	27,215 (32)	104,781 (34)	485,317 (31)
2011–2012	166,134 (68)	86,133 (58)	17.718 (24)	64,041 (28)	334,026 (53)
2012–2013	148,456 (58)	74,378 (43)	15,547 (39)	36,188 (42)	274,569 (43)
2013–2014	162,578 (64)	85,356 (54)	16,567 (27)	33,146 (22)	297,647 (48)
2014–2015	147,345 (64)	75,478 (58)	15,467 (25)	36.664 (32)	274,954 (54)
2015–2016	106,496 (51)	58,925 (47)	12,576 (32)	33,133 (39)	211,130 (34)
Total	1587,517 (66)	836,294 (49)	177,012 (31)	591,056 (29)	3,191,879 (48)

## 4 DISCUSSION

Retrospective studies of infections encountered at slaughterhouses provide useful prevalence estimations which can be used in risk assessment or future planning of animal diseases’ control and prevention programmes. Few studies conducted economic analyses of these infections in slaughtered animals in Iran (Borji, Azizzadeh, & Kamelli, 2012; Borji & Parandeh, 2010). To the best of our knowledge, there are no other reports studying the monetary losses due to parasitic infections in this Province.

The rate of *E. granulosus* infection estimated in the current study (3.9%) was lower than previous reports from the same sub‐region (5.3%) (Nabavi et al., 2014) and from the north and central Khorasan Province (4.1 and 6.7%, respectively) (Borji, Azizzadeh, & Afsai, 2012; Jahed Khaniki et al., 2013). The rate of *E. granulosus* is also lower than that from other regions of Iran such as Northwest (9.6%) (Mirzaei et al., 2015), West (6.3%) (Chalechale et al., 2016), Central (6.5%) (Azami et al., 2013) and Southern regions (11.6%) (Oryan et al., 2012). The rate was, however, higher than in Khuzestan Province, southwest Iran (3.1%) (Borji, Azizzadeh, & Kamelli, 2012). Moreover this rate was even lower than rates reported from other countries such as Saudi Arabia (8.1%) (Ibrahim, 2010), Northern Greece (4.8%) (Founta et al., 2016), Algeria (13.9%) (Laatamna et al., 2019), Chile (21.1%) (Stoore et al., 2018) and higher than Karbala, in neighbouring Iraq (1.8%) (Jawad et al., 2018). The results revealed that the rate of liver and lung HC infection decreased between 2008 and 2009.

There was a significant seasonal variation of liver and lung rate condemnation due to HC. Seasonal differences may be due to seasonal variation of animal ages since echinococcosis is a chronic infection with cattle remaining infected for the rest of life or a possible gradually degeneration of cysts.

Several studies showed that the HC is a growing concern for public health, as it is considered as an emerging/reemerging infection in several regions of the world. Previous studies (Mahmoudi et al., 2019) estimated the prevalence of HC in Iranian adult humans between 1990 and 2017 to 5%.

According to the present study, 3.3% of livers were condemned due to liver flukes. This rate was similar to a study in the north Khorasan Province, East Iran (5.33%) (Borji & Parandeh, 2010) and lower to North Iran (20.1%) (Radfar et al., 2015). In the West of Iran, lower rate was reported (1.5%) (Shahbazi et al., 2016; Abdi et al., 2013). *D. dendriticum* condemnation rate showed a marked fluctuation during the study period that may be due to substandard training of inspectors, rapid slaughter rates and poor meat inspection facilities.

The rate of *Fasciola* spp. is lower in South Eastern Iran than has been reported elsewhere in the world. For instance, in Brazil a rate of 11.9% (Pritsch et al., 2019) was reported in livers of buffaloes slaughtered from 2003 to 2017. This rate was 2.17% in dairy cattle slaughtered during 2015 and 2016 in Portugal (Barbosa et al., 2019). In South Africa, Jaja et al. (2017) reported a rate of 20.3%, while in the southwest region of Cameroon the rate of *Fasciola* spp. was 22.62% (Kouam et al., 2019). In Denmark, a retrospective study through meat inspection records of nearly 1.5 million cattle slaughtered between 2011 and 2013 reported rates of 25.6–28.4 and 29.3% in 2011, 2012 and 2013 (Olsen et al., 2015). A survey in Lublin Province slaughterhouse, Poland, revealed a rate of 11.97% in hepatic fasciolosis between 2009 and 2012 (Kozlowska‐Loj & Loj‐Maczulska, 2013).

Liver condemnation due to *Fasciola* spp. was lower in winter. The risk of fasciolosis is determined by the density of infected lymnaeid snails in the grazing area. The disease has a predictable seasonal pattern in regions where snails are active for only part of the year. In particular, temperature and rainfall affect both their spatial and temporal abundance and the rate of development of fluke eggs and larvae. Moreover the availability and the number of metacercariae accumulating on herbageis determined the timing and severity of hepatic fasciolosis (Radostits et al., 2007).

The rate of *T. saginata* cysticercus in the slaughtered cattle investigated in the present study (0.13%) was lower than those reported in other regions of Iran (0.25%) (Khaniki et al., 2010; Oryan et al., 2012) but higher than earlier reported from north Khorasan Province (0.004%) (Borji & Parandeh, 2010). While, *Taenia asiatica* have never been reported in Iran and are unlikely endemic as a result of religious prohibitions of pork meat, infections with *T. saginata* persist. The adult *T. saginata* present in the intestinal lumen of the final human host, is commonly characterized by minor, or absence of clinical symptoms, rare complications such as mild diarrhoea, abdominal uneasiness and acute appendicitis have been described. Globally, the rate of bovine *T. saginata* cysticercus by routine meat inspection was estimated to 0.2% in Belgium (Jansen et al., 2018), 0.6% in Brazil (Rossi et al., 2017) and 3% in Rwanda (Nzeyimana et al., 2015). Additionally, bovine *T. saginata* cysticercus in the Middle East and North Africa based on meat inspection was identified in Egypt and Israel, with occurrence ranging from 0.2% to 20% (Saratsis et al., 2019). There is a need for a more thorough investigation, including carcass dissection and immunological tests (Mushonga et al., 2018), to come up with a more accurate rate of this parasite as routine meat inspection may underestimate the true infection rate. Moreover the present study showed that carcasses condemnations for *T. saginata* cysticercus were higher in summer. Bovine *T. saginata* cysticercus is a result of the ingestion of the Taenia's eggs, usually in contaminated herbage through exposure to human faeces. So, the transmission of *T. saginata* cysticercus is low in the absence of dew, rain, or irrigation. Outbreaks of Taenia's eggs in the pasture may occur after the first rains in spring. Consequently, bovine *T. saginata* cysticercus may be more common in late spring (Taylor et al., 2016). The absence of *Sarcocystis* cysts based merely on macroscopic inspection by meat inspectors cannot be trusted as this parasite has been reported in cattle slaughtered in Tabriz, northwest Iran (8.2%) (Mirzaei & Rezaei, 2016).

Parasites were responsible for 6.4% of organ/carcass condemnations prejudicing the farmers in the study are the direct monetary cost to U.S. $3,191,879 during the 8‐year period (20 April 2008 to 19 March 2016). This figure is gross underestimation of the real total financial losses as it did not take in account several indirect losses such as reduced productivity, costs of veterinary care as well as deaths (Perry & Randolph, 1999). Consequently, even though the financial loss due to condemnation of carcasses and organs (lungs and livers) due to parasitic lesions was high, the total impact of parasites on the region's livestock industry is higher than what was estimated in the current study. Moreover it should be noted that the rate of parasitic infections based on retrospective studies in slaughterhouses are certainly underestimated and the economic impact of these infections, some of them were present but the veterinarians did not condemned the organs.

## 5 CONCLUSION

The estimate of the financial loss is, almost certainly, a gross underestimate of total financial losses as it takes no account of the premature deaths, low body weights and sub‐optimal milk that can result from parasitic infections. Based on organ condemnation rates observed in this study, implementation and periodic review of routine livestock infection surveillance systems, including an effective trace‐back system, are recommended. This will help to reduce the burden of infections in animals. However, further studies are needed to obtain a complete overview of the epidemiology of these parasitic infections in other definitive hosts, in order to implement control and preventive measures specifically targeting farms in the studied region.

## CONFLICT OF INTEREST

The authors declare that there is no competing interests.

## AUTHOR CONTRIBUTIONS

**Javad Khedri:** Data curation; Investigation; Methodology. **Mohammad Hossein Radfar:** Data curation; Investigation; Methodology. **Behzad Nikbakht:** Data curation; Investigation; Methodology. **Rohollah Zahedi:** Data curation; Investigation; Methodology. **Mehdi Hosseini:** Data curation; Investigation; Methodology. **Mohammad Azizzadeh:** Formal analysis; Software; Validation. **Hassan Borji:** Conceptualization; Funding acquisition; Investigation; Methodology; Project administration; Supervision; Validation; Visualization; Writing‐original draft; Writing‐review & editing.

## ETHICS APPROVAL AND CONSENT TO PARTICIPATE

With regard to what constitutes appropriate ethics and consent/animal welfare statements, Retrospective studies did not require ethics committee.

### PEER REVIEW

The peer review history for this article is available at https://publons.com/publon/10.1002/vms3.475.

## Data Availability

The datasets used and/or analysed during the current study available from the corresponding author on reasonable request.
